# Supplementary material for: Wafer-Scale Room-Temperature Bonding of Smooth Au/Ti-Based Getter Layer for Vacuum Packaging
Source: Sensors (Basel). 2022 Oct 24;22(21):8144. doi: 10.3390/s22218144 (PMC9658547; doi:10.3390/s22218144)
Supplement: Supplementary file 1 [file sensors-22-08144-s001.zip › sensors-1944185-supplementary.pdf]

Supplementary Materials

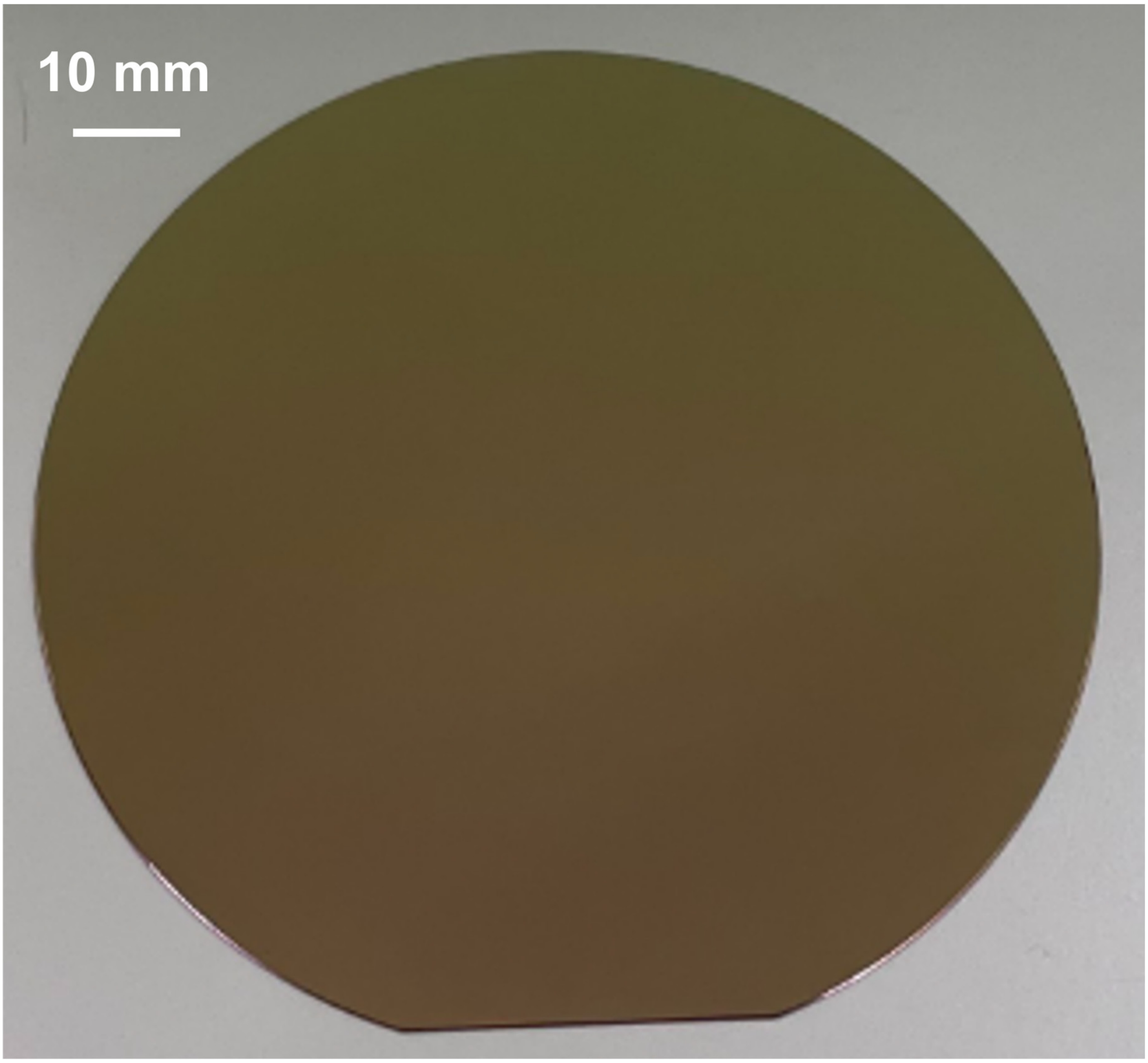

Figure S1. Thermally oxidized Si wafer having a 300-nm-thick SiO_2 layer used as Template wafer.
